# Representation of Auditory Task Components and of Their Relationships in Primate Auditory Cortex

**DOI:** 10.3389/fnins.2020.00306

**Published:** 2020-04-21

**Authors:** Stanislava Knyazeva, Elena Selezneva, Alexander Gorkin, Frank W. Ohl, Michael Brosch

**Affiliations:** ^1^Leibniz Institut für Neurobiologie, Magdeburg, Germany; ^2^Institute of Psychology, Russian Academy of Sciences, Moscow, Russia; ^3^Institute of Biology, Otto-von-Guericke University, Magdeburg, Germany; ^4^Center for Behavioral Brain Sciences, Otto-von-Guericke University, Magdeburg, Germany

**Keywords:** classical conditioning, instrumental conditioning, agency, monkey, sound (audio) processing

## Abstract

The current study aimed to resolve some of the inconsistencies in the literature on which mental processes affect auditory cortical activity. To this end, we studied auditory cortical firing in four monkeys with different experience while they were involved in six conditions with different arrangements of the task components sound, motor action, and water reward. Firing rates changed most strongly when a sound-only condition was compared to a condition in which sound was paired with water. Additional smaller changes occurred in more complex conditions in which the monkeys received water for motor actions before or after sounds. Our findings suggest that auditory cortex is most strongly modulated by the subjects’ level of arousal, thus by a psychological concept related to motor activity triggered by reinforcers and to readiness for operant behavior. Our findings also suggest that auditory cortex is involved in associative and emotional functions, but not in agency and cognitive effort.

## Introduction

It is well established that neuronal activity in auditory cortex not only reflects sounds but also other components of auditory tasks, including stimuli of other sensory modalities if relevant for the task, motor actions that are executed relative to sounds, as well as the reinforcers that motivate subjects to perform acoustically-guided actions (Scheich and Brosch, 2013). In addition, neuronal activity during and in between task components reflects the mental processes that are required to accomplish a given task. It has been reported that sound-evoked activity varies with the motor response that has to be executed after sounds (*sensorimotor association*; Vaadia et al., 1982; David et al., 2012; Jaramillo et al., 2014; Huang et al., 2019), differs between conditions in which sound is self-generated by the subject’s own behavior and in which it is externally generated (*sense of agency*; Eliades and Wang, 2003; Otazu et al., 2009; Carcea et al., 2017), depends on reinforcers that are received for correct sensorimotor behavior and on unconditioned stimuli that automatically follow conditioned sound stimuli (*value of sounds*; Kitzes et al., 1978; Rutkowski and Weinberger, 2005; Letzkus et al., 2011; David et al., 2012), depends on the cognitive *efforts* the animals deploy on sound processing (Fritz et al., 2003; Atiani et al., 2009; Kuchibhotla et al., 2017), and is related to the general activity level of animals (*arousal;* Schneider et al., 2014; Zhou et al., 2014; McGinley et al., 2015; Williamson et al., 2015), working memory load (Huang et al., 2016), sound and reward expectation (Selezneva et al., 2006; Brosch et al., 2011b), and the types of cognitive operations that are performed on sounds (Fritz et al., 2003; Selezneva et al., 2006; Brosch et al., 2015).

Although the cited studies agree in that different mental processes affect neuronal activity in auditory cortex, there appears little consensus in how they change this activity, e.g., whether neuronal firing rates increase or decrease when the value of sounds change. In addition to the cited studies, there are also several studies reporting that neuronal activity in auditory cortex did not change across auditory tasks (e.g., Hocherman et al., 1976; Gilat and Perlman, 1984; Abolafia et al., 2011) or when sounds are differently associated with reinforcers (Diamond and Weinberger, 1989).

We argue here that there are different reasons for the divergent findings in the cited studies. Firstly, some studies the conditions compared with one another differed in more than one aspect of an auditory task. For example, when a sound detection task is contrasted with passive stimulation, the two conditions differed both in whether motor responses were executed and in the presence of reinforcement (Fritz et al., 2003; Bgur et al., 2018). Thus, more conditions are required to disentangle motor from reinforcement effects. Secondly, some studies did not take into account that neuronal activity could be related to non-auditory task components. Thus, it was not clarified whether changes in the sound-evoked activity were “true” task-related effects or solely related to motor behavior or to reinforcers (e.g., Eliades and Wang, 2003). Thirdly, some studies did not consider that different levels of tonic firing between task components may have obscured or created differences in sound-evoked activity between conditions (e.g., Carcea et al., 2017). Fourthly, it is often not possible to disambiguously relate differences between experimental conditions to specific mental processes. For example, a condition with self-generated vocalizations and a condition in which recorded vocalizations were played back by a computer differed not only in agency but also in the predictability of sounds (Müller-Preuss and Ploog, 1981; Eliades and Wang, 2003). Finally, conditions were tested in animals with different training experiences on the relationships of the task components (Selezneva et al., 2017) and thus that differences in sound-evoked activity could also be due to differences in experience.

**FIGURE 1:**
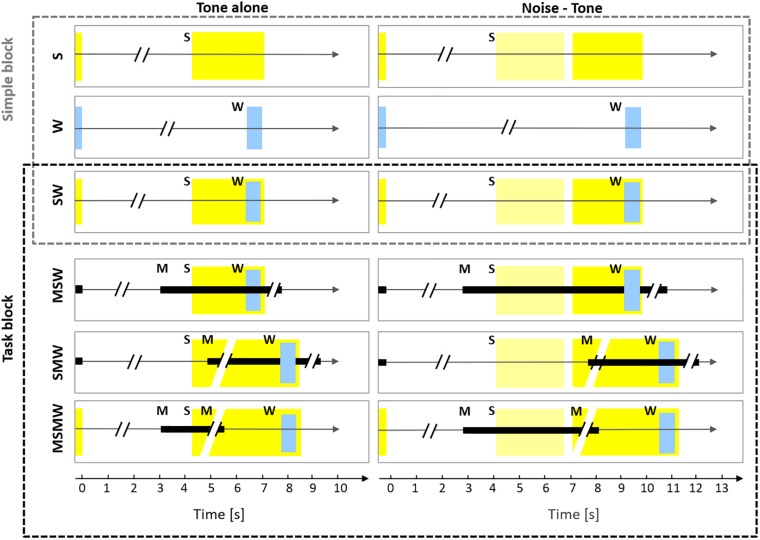
Experimental design. The six rows show the six conditions used in this study which differed in the number and order of the task components S (sound), W (water), and M (motor response). The left column shows conditions in which only a tone was presented (tone alone) and the right column shows conditions in which a noise burst and a tone were presented (noise-tone). The first row depicts the S-condition in which a trial consisted only of sound, either a tone (yellow rectangle; 2.2 s duration) or a noise burst (pale yellow rectangle; 1.6 s duration) followed by the tone. The silent idle period between the sounds presented in consecutive trials varied between 3.5 and 7 s. In the W-condition, only water (W; blue rectangle) was delivered for 0.6 s in a trial and the idle period between consecutive trials varied between 5 and 10.3 s (for the sake of presentation the W-condition is shown in both columns). In the SW-condition, a trial consisted of sounds and water, the latter was delivered from 1.5 to 2.1 s after tone onset. The SW-condition was tested both in the Simple block (upper three conditions) and in the Task block (lower three conditions). In the MSW-condition, the monkeys themselves initiated a trial by bar touch. While holding the bar (black rectangle), sounds were presented and water was delivered. In the SMW-condition, a trial was externally initiated by a computer and started with sounds, in response to which the monkeys had to touch the bar at the earliest 0.3 but not later than 1.2 s after the onset of the tone. After holding the bar for 1.5 s, water was delivered. The monkeys had to release the bar and remain idle for 3.5–7 s before the next trial. In the MSMW-condition, the monkeys initiated a trial by bar touch. While holding the bar, sounds were presented. The monkeys had to release the bar from 0.3 to 1.2 s after the onset of the tone, which was rewarded with water 1.5 s after release.

The current study aimed to resolve some of the inconsistencies in the literature on which mental processes affect auditory cortical activity. To this end, we recorded neuronal firing from the primary auditory cortex of monkeys while they were involved in six conditions differing in the arrangement of the task components sound, motor action, and water reward (Figure 1). Conditions were designed such that comparison across conditions allowed to study the involvement of auditory cortex in various mental processes, including but not limited to sensorimotor association, sense of agency, effort and arousal. Three of the conditions were simple: one with sound presentations only (*S-condition*; either a tone or a noise burst followed by a tone), one with water delivery only (*W-condition*), and one in which water was delivered after the tone (*SW-condition*). These conditions were complemented by three task conditions. Here, specific motor behavior was required either before, after, or both before and after the sounds in order to receive the water reward. In the *MSW-condition*, subjects had to exhibit motor behavior (touching a bar) to generate sounds. In the *SMW-condition*, subjects had to exhibit motor behavior after the presentation of the tone. In the *MSMW-condition*, subjects had to exhibit two motor behaviors, one to generate sounds (touching a bar) and another to the tone (releasing the bar). We note that the MSMW-condition and the MSW-condition were self-initiated conditions and the remaining conditions were externally initiated conditions. We also note that the MSMW-condition and the SMW-condition required higher efforts than the corresponding conditions in which no such motor responses were required (the MSW-condition and the SW-condition). Comparisons between pairs of conditions which differed in the presence (or absence) of one component allowed us to identify which arrangement of task components affected the firing in auditory cortex and also to make inferences on the mental processes to which auditory cortex contributes. For example, comparison between the S-condition and the SW-condition helps to clarify whether neuronal activity in auditory cortex provides a correlate of the value of sounds. This and other comparisons were also used to address the question whether auditory cortex activity is related to audio-motor associations, the sense of agency, effort, and arousal. Because the comparisons were carried out on the same neuronal populations we could also determine the relative effect size of the different mental processes on the neuronal firing.

We trained two monkeys until they were able to perform all three tasks, to rapidly switch between them, and to be idle during the three simple conditions within a single experimental session. It is possible that the task-related neuronal activity identified in these instrumentally-trained monkeys differs from that of monkeys with different training experience on the same task components. To address the effects of experience on auditory cortical activity, we also tested two other monkeys on the three simple conditions only.

## Materials and Methods

### Subjects

Experiments were performed on adult female (monkey B and monkey E) and male (monkey W and monkey D) long-tailed macaques (Macaca fascicularis). More than 1 year before initiation of the current study, the two male monkeys had participated in experiments in which they performed auditory tasks different from those used here (Brosch et al., 2015; Lovell et al., 2015). The two female monkeys were naïve prior to the initiation of the current study. Monkey B and monkey W will be referred to as the instrumentally-trained monkeys, and monkey E and monkey D as the passive monkeys.

Under deep anesthesia [Ketamine HCl (4 mg/kg) and Xylazine (5 mg/kg)], a headholder (“halo”) device was implanted onto the monkeys’ head to allow atraumatic head restraint (details in Brosch and Scheich, 2008). It consisted of three strong arches that closely encircled the occipital, supra-orbital and mid-sagittal ridges of the head. This helmet-like piece was firmly and permanently attached to the head by means of several counteracting stainless steel bolts with sharpened points, which were advanced by rotation through the intact skin and soft tissue until they lodged firmly against the skull. Subsequently, monkeys received a chamber implant; monkey W and monkey B on the right side and monkey E and monkey D on the left side. For the implantation, a piece of bone was removed with a trephine (diameter: 21 mm) and an externally threaded stainless steel cylinder was screwed into the slightly undersized hole. The operations were followed by a full course of antibiotic (Pyanosid or Bytril, 0.2 ml/kg) and analgesic (Carprofen, 0.1 ml/kg) treatment during which animals were monitored several times on a day.

Throughout the experiments, the monkeys were housed in groups of two or three animals in individual cages, in which they had free access to dry food. They earned a large proportion of their water ration during the daily training sessions and received the remainder in the form of fresh fruit during and after each session, and during the weekends.

All experiments were approved by the Animal Care and Ethics Authority of the State of Saxony-Anhalt (Landesverwaltungsamt Halle) and conformed to the EU Directive (2010/63/EU) on the protection of animals used for scientific purposes. We support the principles of the consortium on Animal Research Reporting of *In Vivo* Experiments (ARRIVE).

### Apparatus

Experiments were carried out in a double-walled soundproof room (1202-A, IAC). For all behavioral training and testing, a monkey squatted in a primate chair. Its front compartment accommodated three light-emitting diodes for visual stimulation (LED, each 2 visual degrees in diameter), a touch bar, and a spoon which could be filled with small amounts of fluids for reinforcement.

A computer was used to generate visual and auditory stimuli, to monitor touching of the bar, and to control the delivery of fluids to a monkey. Auditory stimuli were either generated with a waveform generator (WG1, Tucker-Davis Technologies) or with an array processor (AP1, Tucker-Davis Technologies), interfaced with the computer. The output of the two devices was amplified (Pioneer, A202) and presented through two loudspeakers (Karat 720, Canton) which were placed at a distance of ∼1 m and at an angle of 40° on the left and the right side of the monkey (also see Rahne et al., 2008).

As reinforcers, or unconditioned stimuli, we used small amounts of fluid (0.3–0.8 ml), either in form of drinking water or of a thin smoothie (made from pureed raw fruit and vegetables and diluted with water). This fluid was deposited on the spoon by means of a pump (SP200IZ, World Precision Instruments) equipped with a plastic syringe. The spoon was located in front of the monkey’s mouth such that the monkey had to actively lick the water with their tongue from the spoon.

The monkeys were monitored with a digital camera (ICD-848P, Ikegami). In some of the experimental sessions the video signal was recorded into a computer, using Pinnacle Studio software (25 frames per second).

In most sessions, we used a multichannel drive (Mini Matrix, Thomas Recording) equipped with up to five micro-electrodes (impedance 1.5–2.5 MΩ) and a built-in pre-amplifier to record the action potentials fired by a small group of neurons from different sites of auditory cortex. The drive was attached to the monkeys’ headholder through a rigid connecting piece. In about half of the sessions of monkey W, we used a single microdrive (MEM 10, Thomas Recording) which was directly clamped to the recording chamber by means of a custom-made adaptor. The adaptor also allowed x-y-z positioning of the microdrive. The microdrive was equipped with a multitrode (300 μm diameter; Thomas Recording) with seven gold contacts (each with a diameter of 40μm and an impedance of 1.5–1.9 MΩ) and with a spacing of 125 μm between the contacts. An eighth contact was on the tip of the multitrode (impedance 0.5 MΩ), 700 μm from the first of the other contacts. The signals on the multitrode were pre-amplified (PA-08, Thomas Recording). In sessions in which the microdrive was directly clamped to the recording chamber, the head of the monkey was not rigidly fixated but only partially restrained by surrounding the head with a custom-made box-like device made of plexiglass which was tightly fitted around the head. Based on the spatial distribution of best frequencies of the multiunits at different sites and the depth relative to cortical surface we inferred that most sites were located in the primary auditory cortex.

Following preamplification, the signals from each electrode were amplified and filtered at 0.5–5 kHz with a PGMA-64 (Thomas Recordings). The action potentials of small groups of neurons (multiunits) were isolated with an A/D data acquisition system (Cheetah32, Neuralynx), and their time stamps and waveforms were sampled at a rate of 44.1 kHz.

### Rationale and Experimental Conditions

We generated six experimental conditions which differed in the number and the order of the components sounds (S), motor behavior (M), and water (W) (Figure 1). We limited the number of conditions to six to allow for multiple task switching within a single sessions while allowing to collect a sufficient number of trials per task condition. Two of the six conditions contained a single component only; these were the *S-condition* with sounds only and the *W-condition* in which only water was delivered. The M-condition was not implemented because the monkeys would not execute the same hand movement over and over again without being reinforced with water. The only two-component condition was the *SW-condition*, in which sounds were followed by water. Motor behavior was only required in the remaining three multi-component task conditions. In the MSW-condition, subjects had to exhibit a specific motor behavior (touching a bar) to generate sounds and to receive water. In the SMW-condition, subjects had to exhibit specific motor behavior after the presentation of sounds to receive water. In the MSMW-condition, subjects had to exhibit two motor behaviors, one to generate sounds (touching a bar) and another to the sounds to receive water (releasing the previously touched bar). We note that the MSMW-condition and the MSW-condition were self-initiated conditions and the remaining conditions were externally initiated conditions. We also note that the MSMW-condition and the SMW-condition required higher efforts than the corresponding conditions in which no such responses were required to the tones (the MSW-condition and the SW-condition). The three task conditions were also used to estimate neuronal activity related to motor behavior alone.

As S-component we used two sounds (a pure tone and a noise burst), instead of one, because we wanted the monkeys to perform a sound discrimination task, and not a sound detection task, and thus to involve them in a task that required auditory cortex (Heffner and Heffner, 1986). The use of two sounds also excluded the possibility that the monkeys relied on other cues for their motor responses, including the timing cue. Two sounds rather than one sound of variable duration also had the advantage that we could test task effects on different types of sounds. The frequency of the tone varied across experimental sessions between 0.4 and 10 kHz but was constant within an individual session and adjusted to the frequency sensitivity of the neurons under investigation. The sound pressure level was 60 dB SPL. In three conditions (the S-condition, the SW-condition, and the MSW-condition), the tone had a fixed duration of 2200 ms. In the two conditions in which the monkeys had to exhibit a motor response to the sounds (the SMW-condition and the MSMW-condition), the duration of the tone was varied between 2500 and 3400 ms to account for the variable reaction times. The noise burst always was same, with fixed spectro-temporal characteristics, a duration of 1600 ms and a sound pressure level of ∼60 dB SPL. In individual trials, we presented either only the pure tone (tone-alone trial) or a noise burst followed by the pure tone after a silent interval of 200 ms (noise-tone trial). Both sounds had a relatively long duration so that the neuronal responses to the sound transients had minimal overlap with the neuronal activity related to other task components and that there was a sufficiently long period to identify and analyze tonic activity.

As the M-component, we considered two types of motor behavior of the monkeys. They either touched and held the bar for some time (in the MSW- and SMW-condition and at the first M-component of the MSMW-condition), or they released the bar (at the second M-component of the MSMW-condition).

As the W-component, a small amount of fluid was delivered for a period of 600 ms onto a spoon which was located very close to the monkeys’ mouth and where the fluid remained until it was licked off by the monkeys.

In the S-condition, we presented either the tone with a duration of 2200 ms, or the noise burst with a duration of 1600 ms followed by the tone. The sounds were followed by an *idle period* of variable duration (3.5–7.0 s) before the sounds of the next trial were presented. Generally, the idle period was defined as the time between the end of the last component in the current trial and the beginning of the first component in the subsequent trial. In the S-condition and other simple conditions, every bar touch restarted the idle period to discourage such behavior. In the W-condition, only water was delivered and this was followed by an idle period, which varied randomly between 5.0 and 10.3 s. In the SW-condition, water was delivered from 1500 to 2100 ms after tone onset, both in tone-alone trials and in noise-tone trials. The idle period varied between 3.5 and 7.0 s.

In the MSW-condition, the monkeys received water for holding the bar for some minimal period of time, during which also the sounds were presented. In tone-alone trials, the tone started 750 ms after bar touch and the minimal hold period was 2250 ms. In noise-tone trials, the noise burst started 1050 ms after bar touch and the minimal hold period was 4350 ms. The different delays between the M- and S-component in tone-alone and noise-tone trials were accidentally implemented. In both trial types, water was delivered from 1500 to 2100 ms after tone onset to have the same temporal relationship between the sounds and the water as in the SW-condition. After tone offset, the monkeys had to release the bar and stay idle for at least 4000 ms before they could start the next trial by touching the bar. Br releases during the minimum hold period immediately stopped the sounds and the trial was scored as mistake. Br touches during the idle period reset the minimum idle time to 4000 ms, thus providing a mild form of punishment.

In the SMW-condition, a tone was presented for maximally 3400 ms, either alone or 200 ms after the presentation of the noise burst. The monkeys had to discriminate the noise burst from the tone by holding the bar for a period of 1500 ms at the earliest 300 ms but not later than 1200 ms after tone onset. If they did so, the trial was scored correct and water was administered. The tone stayed on during the hold period and during water delivery and was turned off 100 ms after water delivery, as in the SW-condition. Br touches earlier than 300 ms after tone onset as well as bar releases during the 1500 ms hold period, immediately stopped the sounds and were scored as mistake (false alarm). Br touches later than 1200 ms after tone onset were also scored as mistake (miss). After sound offset, the monkeys had to release the bar and stay idle for 3500–7000 ms before the next trial started with sounds.

The MSMW-condition was initially identical to the MSW-condition and thus was also a self-initiated condition. Thus, the monkeys had to hold the bar which either triggered the tone after 750 ms (in tone-alone trials), or triggered the noise burst after 1050 ms, which was followed by the tone (in noise-tone trials). The remainder of the trial was identical to the SMW-condition, i.e., the monkeys had to release the bar from 300 to 1200 ms after the onset of the tone to receive the water reward. After sound offset, the monkeys had to stay idle for at least 4000 ms until they could start a new trial by bar touch.

We defined the trial beginning as the moment in time at which the first component of a condition commenced. This was the bar touch in the MSMW- and MSW-conditions, the sound in the SMW-, SW-, and S-conditions, and the water in the W-condition.

We divided the training of the monkeys on the three task conditions into five stages, in each which the monkeys remained until they performed correctly in more than 70% of the trials for more than 3 consecutive training sessions. (1) Initially the monkeys were trained for 2 months on the MSW-condition. During the subsequent training stage. (2) The monkeys were trained over a period of 1 month to perform the MSMW condition. (3) After having learnt to perform the MSW condition and the MSMW condition the monkeys were trained for 2 months to be able to switch between the two tasks within the same training session. During training stage 4, the monkeys were trained for 1 month to perform the SMW condition. This training was followed by training stage 5, in which the monkeys learnt over period of 2 months to switch between the three tasks within the same training session.

During an individual experimental session the different conditions were tested in separate blocks, each consisting of 60–160 trials. In the instrumentally-trained monkeys, we initially tested the three task conditions together with the SW-condition in random order that was counterbalanced across sessions. In some of the sessions, the four conditions of this *task block* were followed by the *simple block* in which we tested, in random order, the SW-condition again together with the other two simple conditions. In the passive monkeys, only the three simple conditions were tested. The currently active condition was indicated by illuminating one or two of the three LEDs located in front of the monkeys. Although we noticed that the monkeys were clearly alerted when the illumination of the LEDs changed, it is unclear whether they used the information provided by the LEDs. The monkeys rather appeared to gain knowledge about the currently active condition by constantly monitoring the task contingencies. This monitoring likely resulted in a constantly increased level of general attention despite individual conditions not being highly demanding.

At the beginning and at the end of a session, 400 pure tones with 40 different frequencies were presented at ∼60 dB SPL to assess the best frequency and the tuning curve of each multiunit (Oshurkova et al., 2008). Frequencies were equidistantly spaced on a logarithmic scale over a range of 8 octaves (0.11–27.2 kHz). All tones had duration of 100 ms and were presented in a pseudorandom order with a stimulus-onset interval of 1000 ms until all frequencies had been presented ten times. The data from the two runs of tone presentations were combined for analysis.

### Data Analyses

All data analyses were carried out with custom-written scripts in MatLab (Versions 7.5 and 9.0).

We re-examined the waveforms of all online detected events using a principal component analysis with the aim to identify and further exclude artifactual waveforms from our multiunit recordings.

To determine how the firing rate of a given recording site varied relative to the individual components in the different experimental conditions, we calculated peri-event time histograms (PETHs) with a bin size of 100 ms which were triggered either on sound onset, on the beginning of water delivery, or on bar touch. For each PETH a minimum of 12 trials (mean 49) were averaged. The number of trials used for averaging was lower for time bins after the minimal idle period of the condition. For the task conditions, only correct trials were included. For the simple conditions, only trials with no bar touches were included. The number of error trials was too small to compute interpretable PETHs.

In this report, we used population PETHs computed from all multiunits that were used for a specific comparison across conditions. For each multiunit, we first computed PETHs for each of the conditions tested in an experimental session. We then found the two bins of all PETHs of this multiunit in which the firing rate was minimal and maximal. Subsequently, we subtracted the minimal firing rate from the firing rates in all PETHs and divided this difference through the difference between the maximal and minimal firing rate. In a last step, the normalized PETHs of all multiunits of a specific condition were combined into the median normalized PETH of that condition.

We used Wilcoxon signed rank tests to compare the firing rates in the population PETHs between two conditions in specified time intervals, with a significance level of 0.01. We selected this significance level to account for the multiple comparisons that were performed between conditions. In addition to this analysis across multiunits, we also tested the multiunits individually whether their firing rates differed across trials between pairs of conditions by performing Wilcoxon signed rank tests. Results of these tests revealed the fraction of multiunits with a difference for specific conditions and allowed to draw the same conclusions as those that are made in this report based on the population analysis (see tables) and, for this reason, are not shown here.

The contrast of the firing rates in a given time interval between two conditions was calculated by subtracting the firing rate of the condition with the smaller number of components from the firing rate of the condition with the larger number of components and dividing this difference by the firing rate of the first condition; contrasts are expressed in percent.

All recorded videos were analyzed to detect licking activity (protruding of the tongue, lip folding, smacking and other movements). Using MatLab, we first extracted one frame from the video where the monkey did not make any mouth movements for several seconds. In this resting frame, the coordinates of the “mouth area” with a size of 60 by 50 pixels were determined, which covered the snout of the monkey and the end of the spoon with the deposited water. These coordinates were used to mark the “mouth area” in all other frames from the video. We calculated the averaged difference in the pixel intensities of the mouth area in the resting frame to those in every other frame of the video. The resulting curve was filtered using a rational transfer function with a window size of 400 ms to remove rapid changes. The filtered curve was squared and then, similar to the PETHs, was normalized by first finding the two bins in the curves of all conditions tested in an experimental session in which the licking activity was extreme. Subsequently we subtracted the lowest licking activity from the licking activity in all conditions and divided this difference through the difference between the largest and lowest licking activity. In a last step, the normalized curves of individual sessions were combined into the median licking activity of all available sessions in the monkeys under consideration.

## Results

### The Instrumentally-Trained Monkeys Differentiated and Performed Six Experimental Conditions

Two monkeys (monkey W and monkey B) were trained until they were able to differentiate the six conditions and to perform the three tasks within the same experimental session (Figure 1).

**FIGURE 2:**
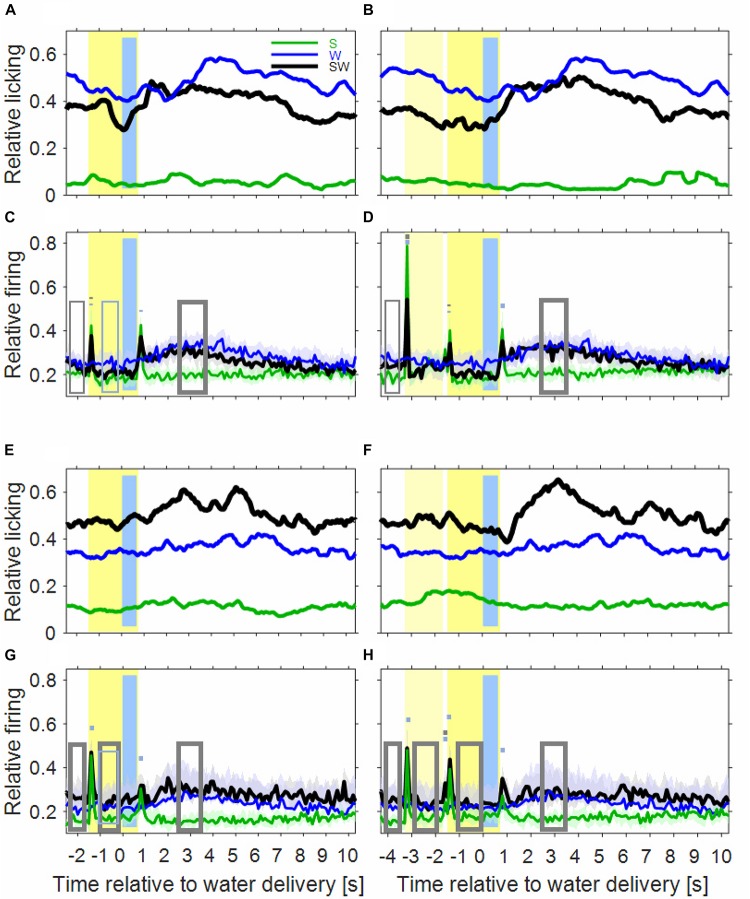
Licking activity and auditory cortical firing rates differ between the three conditions of the simple block. In the first row, the plots show the licking activity of six experimental sessions in the instrumentally-trained monkey B for the S-condition (green), the W-condition (blue), and the SW-condition (black) in tone-alone trials **(A)** and in noise-tone trials **(B)**. Note that the blue curves are identical in tone-alone and noise-tone trials already from 2.5 s before water delivery. Other conventions as in Figure 1. **(C,D)** Show the median population firing rates of 97 multiunits in the two instrumentally-trained monkeys. The shadings show the 95% range of the median. The rectangles and the short bars denote time intervals in which there was a significant difference in firing rates between the S and SW condition (gray) or between the W and SW condition (blue). Thin lines indicate *p* < 0.01; thick lines indicate *p* < 0.001. **(E,F)** Show the licking activity of fourteen sessions in the two passive monkeys. **(G,H)** Show the population firing rates of 75 multiunits in the two passive monkeys.

Inspection of the licking movements by which the monkeys drank the water indicates that the monkeys differentiated the three conditions of the simple block. This is depicted in Figures 2A,B for the licking activity that was derived from the video recordings of six sessions of monkey B. The baseline licking activity in the 1000 ms time interval before sound presentation, or before water delivery, was lowest in the S-condition (green curve), higher in the SW-condition (black curve) and highest in the W-condition (blue curve). The three conditions also differed in how licking activity varied relative to the sounds and the water. In the S-condition, there was no clear relationship between them. In the W-condition, the licking activity increased slowly within ∼3 s after water delivery. In the SW-condition, and particularly in trials in which the noise burst and the tone were presented (*noise-tone trials*), the licking activity decreased slowly from shortly after the beginning of the first sound until the water was delivered. Subsequently, the licking activity increased and peaked 1–2 s after water delivery, after which it slowly decreased to baseline. These differences were described by determining, by linear regression, the 1000 ms interval where the licking activity increased most strongly. This revealed that the strongest increase occurred earlier after water delivery in the SW-condition (1100 ms in tone-alone trials and 820 ms in noise-tone trials) than in the W-condition (2840 ms). This indicates that the monkeys associated the (conditioned) auditory stimulus with the (unconditioned) water stimulus.

For the task block, analysis of the bar touches and releases obtained in 2861 trials from six sessions (three from each monkey) showed that the monkeys readily switched between the three task conditions and the simple SW-condition and that they performed correctly in 92–99% of the trials in each task. Failures resulted from inappropriately handling the bar: The monkeys missed to touch the bar after tone presentation in the SMW-condition or missed to release it in the MSMW-condition; or they prematurely released the bar in the MSW-condition. We also noted that in 6–15% of the trials of the conditions, the monkeys touched the bar during the idle period between trials. The different errors suggest that in some trials the monkeys confused the four conditions and that in a given condition, the monkeys actively suppressed motor behavior that was appropriate for the other conditions. The monkeys likely suppressed such motor behavior also during the simple block as indicated by occasional bar touches.

### Neuronal Activity in the Instrumentally-Trained Monkeys During the Simple Block

To more easily understand how combining task components affects auditory cortical activity, we first describe the results obtained from the simple block. We compared the S- and the W-condition with the SW-condition of 65 multiunits recorded during 17 sessions from monkey W and of 32 multiunits recorded during 8 sessions from monkey B. Best frequencies (BF) of these multiunits ranged from 0.12 to 20 kHz (median 1.6 kHz) and bandwidths of the tuning curves (BW) ranged from 0.46 to 7.95 octaves. The firing rate of each multiunit was normalized and then all 97 multiunits were combined into a median population firing rate for different conditions of tone-alone and noise-tone trials (Figures 2C,D). Contrasts of firing rates between conditions in specific time intervals and their statistical significance are summarized in Table 1. We considered contrasts significant if a *p*-value was < 0.01. To increase readability of the text, we report *p*-values exclusively in Table 2. We obtained similar results when we tested individual multiunits for differences in firing rates between conditions (not shown).

**TABLE 1:** Median contrasts of the firing rates between experimental conditions in the instrumentally-trained monkeys.

**Component**		**Time interval**	**Start (ms)**	**End (ms)**	**S vs. SW**	**W vs. SW**	**SW vs. MSW**	**SMW vs. MSMW**	**SW vs. SMW**	**MSW vs. MSMW**
		Baseline	−1700	−700	**21.3**	−9.9	0.0	−**8.6**	**16.5**	6.5
M		Bar touch	−500	200	X	x	**13.4**	6.7	x	**9.5**
		Bar hold – T	300	1000	X	x	−**6.5**	−**10.6**	x	**10.1**
		Bar hold – NT	300	1000	X	x	−**8.3**	−**13.6**	x	5.6
S	(T)	Tone – on	0	100	−**15.5**	**40.0**	−**9.5**	−0.4	−2.5	7.3
		Tone – off	0	100	−11.0	**28.5**	−8.3	−7.1	−7.6	−6.3
		Tone – SS	−1900	−700	11.9	−**19.9**	−**11.9**	8.8	6.3	**31.2**
	(NT)	Noise – on	0	100	−**25.1**	**87.1**	−**6.6**	1.6	−2.7	5.8
		Noise – off	0	100	−12.6	27.4	−7.1	−0.4	−3.1	3.9
		Noise – SS	200	1600	−7.3	−6.8	−**8.5**	−7.0	1.3	3.0
		Tone – on	0	100	−**17.2**	**34.2**	4.0	−1.8	7.9	1.9
		Tone – off	0	100	−15.3	**42.2**	−5.9	−**10.3**	2.8	−2.0
		Tone – SS	−1900	−700	10.3	−11.5	−7.7	11.0	10.1	**32.5**
W		Water – T	2500	3500	**15.9**	−6.1	−2.4	−1.5	3.6	4.5
		Water – NT	2500	3500	**20.0**	−6.7	−0.4	−5.3	5.7	0.5

**Results are given for different time intervals relative to the task components M, S, and W. The start and the end of each time interval are referred to the beginning of a component, except for the baseline which is expressed relative to the first component of a condition, as well as for the tone-SS which is expressed relative to the tone offset. SS refers to the steady-state part of the sounds. For most comparisons, results are given separately for tone-alone trials (T) and for noise-tone trials (NT). 97 multiunits were used to compare the SW-condition and the S-condition as well as the SW-condition and the W-condition. 180 multiunits were used to compare the MSW-condition with the SW-condition, the MSMW-condition with the SMW-condition, the SMW-condition with the SW-condition, and the MSMW-condition with the MSW-condition. Contrasts are expressed as percentage and are calculated as the difference between the condition with the larger number of components and the condition with the fewer components, divided by the firing rate of the latter condition. Bold numbers indicate significant contrasts (Wilcoxon signed rank test, p < 0.01). We only consider contrasts marked with underlines to indicate the presence of effects related to task performance. The other significant contrasts are not interpreted because significant firing rate differences can be explained by the fact that a component was only present in one of the two conditions under investigation.**

**TABLE 2:** *P*-values of the comparisons shown in Table 1.

**Component**		**Time interval**	**Start (ms)**	**End (ms)**	**S vs. SW**	**W vs. SW**	**SW vs. MSW**	**SMW vs. MSMW**	**SW vs. SMW**	**MSW vs. MSMW**
		Baseline	−1700	−700	0.002	0.026	0.915	0.000	0.000	0.067
M		BAR touch	−500	200	x	x	0.000	0.042	X	0.002
		Bar hold – T	300	1000	x	X	0.007	0.000	X	0.001
		Bar hold – NT	300	1000	x	X	0.005	0.000	X	0.172
S	(T)	Tone – on	0	100	0.002	0.000	0.003	0.238	0.722	0.039
		Tone – off	0	100	0.012	0.001	0.143	0.022	0.025	0.026
		Tone – SS	−1900	−700	0.032	0.000	0.000	0.081	0.179	0.000
	(NT)	Noise – on	0	100	0.000	0.000	0.006	0.668	0.469	0.044
		Noise – off	0	100	0.203	0.015	0.306	0.749	0.464	0.381
		Noise – SS	200	1600	0.140	0.188	0.009	0.018	0.828	0.572
		Tone – on	0	100	0.003	0.006	0.225	0.574	0.115	0.320
		Tone – off	0	100	0.054	0.000	0.121	0.004	0.732	0.255
		Tone – SS	−1900	−700	0.121	0.026	0.029	0.058	0.024	0.000
W		Water – T	2500	3500	0.000	0.032	0.218	0.519	0.430	0.059
		Water – NT	2500	3500	0.000	0.108	0.365	0.012	0.085	0.986

#### Neuronal Activity Related to Sound and to Water

As expected, the population firing rate in the S-condition was increased in several time intervals relative to baseline (green curves in Figures 2C,D). Most of these intervals were single 100 ms bins and reflected phasic sound responses to the transients of the sounds, i.e., to the onsets and the offsets of the tone and the noise burst. There was only weak evidence for tonic sound responses, as indicated by the small increase in the firing rate during the steady-state part of the noise burst from 200 to 1600 ms after sound onset.

Extending previous observations (Brosch et al., 2011b, 2015) we observed water-related firing in auditory cortex also in the W-condition (blue curve in Figure 2C). The firing increased above the baseline firing rate ∼1 s after water delivery and then continued increasing for ∼2 s. The firing rate peaked ∼3 s at a level that was ∼25% above baseline and then returned within ∼2 s to baseline. Water-related firing unlikely resulted from electrical or mechanical artifacts because only events were accepted in our micro-electrode recordings whose waveforms were characteristic of action potentials. Also water-related firing was unlikely evoked by the licking sounds related to water intake because water-related firing preceded the monkeys’ licking activity (see below).

#### Neuronal Activity When Sound Was Paired With Water

The time course of the population firing rates in the SW-condition indicates that auditory cortical neurons were activated by both task components but that their activations differed in several intervals from the superposition of the activations in the two single-component conditions (Figures 2C,D).

The first interval was the 1000 ms baseline before trial begin. Here, the firing rate of the SW-condition was between the firing rates found in the two single-component conditions, with a significant firing rate contrast to the S-condition of 21%.

The next intervals corresponded to the sound transients. All phasic sound responses were smaller in the SW-condition than in the S-condition (contrasts 11–25%), which was significant for all onset responses but for no offset response. Differences were observed both in multiunits that were tested with tones close to their BF and in multiunits that were tested with tones more than two octaves from BF. This suggests that pairing sounds with water changed the responses to all tones within the entire tuning curve of a neuron in the same direction (Zhou et al., 2014). Reductions in the phasic sound responses were also observed when their magnitudes were referred to the firing rate immediately before presentation of a sound (“relative sound response”).

The steady-state firing rates both during the sounds and during a corresponding time window of the W-condition differed between the three simple conditions. Similar to the baseline, the steady-state firing rate of the SW-condition laid between those of the two single-component conditions. It tended to be closer to that of the S-condition and, in tone-alone trials, was significantly smaller (20% contrast) than that of the W-condition.

After water delivery the population firing rate of the SW-condition varied in a similar way as that of the W-condition and hence differed from that of the S-condition. From 2500 to 3500 ms after start of water delivery, the firing rate of the SW-condition was larger than that of the S-condition, both in tone-alone trials and in noise-tone trials (16 and 20% contrast).

#### Water-Related Neuronal Activity Was Linked to the Monkeys’ Licking Activity

To find out how the neuronal activity was related to the monkeys’ behavior, we computed normalized cross covariance functions between the firing rate and the licking activity from 1.5 before to 10 s after water delivery for six sessions and 18 multiunits of monkey B (phasic sound responses were disregarded and replaced by the average firing rates in the two adjacent time bins in the PETH). Covariance was smallest in the S-condition (0.41 in tone-alone trials and 0.38 in noise-tone trials) and larger both in the W-condition (0.92) and in the SW-condition (0.73 in tone-alone trials and 0.95 in noise-tone trials). All covariance functions peaked at time shifts different from zero, indicating that the firing rates led the licking activity by several hundreds of milliseconds.

### Neuronal Activity of the Instrumentally-Trained Monkeys in the Task Block

After having established that pairing sounds with water resulted in large changes of auditory cortical firing compared to the unpaired conditions, we examined the task block and analyzed 108 multiunits recorded during 21 sessions from monkey W and 72 multiunits recorded during 17 sessions from monkey B. BF ranged from 0.14 to 20.47 kHz (median 2.84 kHz) and BW from 0.96 to 7.95 octaves. In almost half of the multiunits, the task block was tested before the simple block. We noticed that the population firing rates observed in the SW-condition (which was tested twice) were very similar in the two blocks (not shown).

**FIGURE 3:**
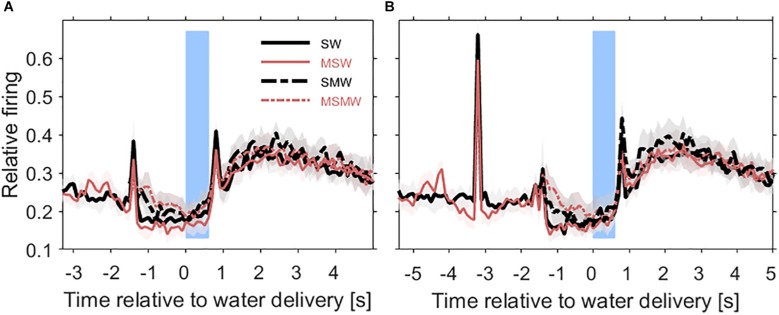
Population firing rates relative to the time of water delivery in the four conditions of the task block. Results are from 180 multiunits in the auditory cortex of two instrumentally-trained monkeys and are shown for the SW-condition (black solid curve), the MSW-condition (red solid curve), the SMW-condition (black dashed curve), and the MSMW-condition (red dashed curve) in tone-alone trials **(A)** and in noise-tone trials **(B)**. Other conventions as in Figure 1. Note that in the SW-condition and MSW-condition, the firing peak ∼1.5 s before water delivery reflected the sound-evoked response. In the SMW-condition and MSMW-condition, this peak reflected the motor response to the tone.

**FIGURE 4:**
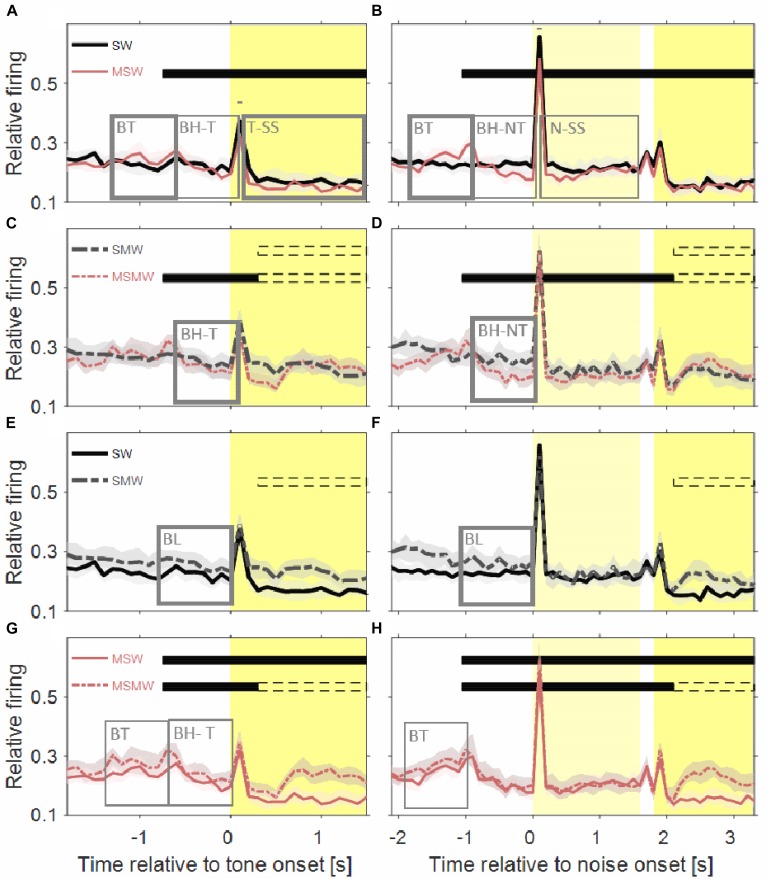
Comparisons of population firing rates between pairs of conditions of the task block. The time is relative to the first sound in a trial. **(A,B)** SW versus MSW, **(C,D)** SMW versus MSMW, **(E,F)** SW versus SMW, **(G,H)** MSW versus MSMW. Other conventions as in Figures 1, 3. The dashed rectangles indicate the time interval in which the monkeys had to exhibit a motor response to the tone. The solid rectangles and the short bars denote time intervals in which there was a significant difference in firing rates between the two conditions shown in a panel, with thin lines indicating *p* < 0.01 and thick lines indicating *p* < 0.001.

Figures 3, 4 show the population firing rates of all 180 multiunits in the three task conditions and in the simple SW-condition, either relative to water delivery or relative to the sound. As summarized in Table 1, the firing rates differed in many intervals between selected conditions, most of which were before tone offset. To find out which of these differences were simply due to the monkeys’ motor actions we estimated the firing related to bar touch by comparing the three task conditions and finding the intervals in which the firing rates did not differ between the tasks. Our experimental design included no condition with motor behavior only.

#### Neuronal Activity Related to Touching and Holding the Bar

**FIGURE 5:**
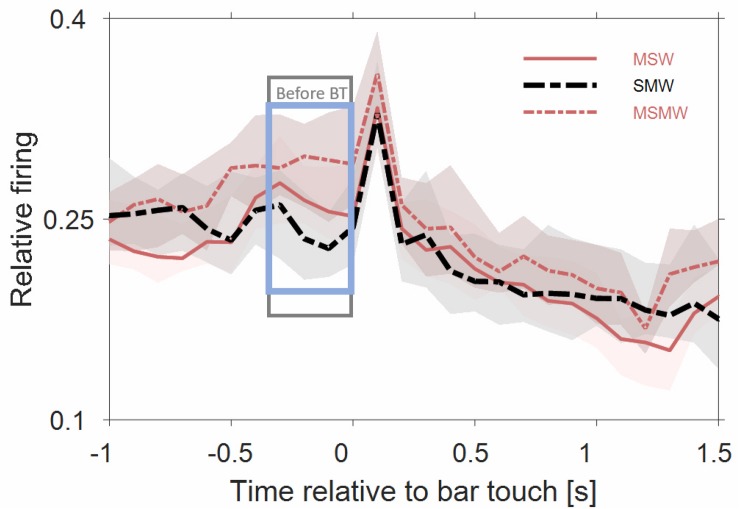
Population firing rates of 180 multiunits in auditory cortex of two instrumentally-trained monkeys relative to bar touch. Conventions as in Figure 3. Data from tone-alone and noise-tone trials were combined. For the two self-initiated conditions, we disregarded the phasic sound responses to the first sound after the bar touch. The rectangles denote time intervals in which there was a significant difference in firing rates between the SMW and MSW condition (gray) or between the SMW and MSMW condition (blue).

Figure 5 shows that the population firing rates of the 180 multiunits differed significantly between the SWM-condition and both the MSW-condition (*p* = 0.008) and the MSMW-condition (*p* = 2^∗^10^–5^) only during the last 300 ms before bar touch but not from 0 to 1000 ms after bar touch (*p* > 0.052). Hence bar touch-related firing appears to consist of a sharp increase in rate shortly after bar touch which is followed by a slow and monotonic rate decrease. Whether this decrease continued beyond 1 s and whether there is also bar touch-related firing before bar touch cannot be clarified with our approach.

#### Neuronal Activity in Externally- and Self-Initiated Conditions

We performed two comparisons between externally-initiated and self-initiated conditions on the same population of neurons, one between the SW-condition and the MSW-condition and another between the SMW-condition and the MSMW-condition. The two pairs of conditions differed in the level of effort that was required to receive water in a trial: it was low in the first pair where the monkeys automatically received water; it was high in the second pair where the monkeys had to execute a motor response to the sounds to receive water.

##### SW-condition vs. MSW-condition.

The population firing rate of the SW-condition differed in several intervals from that of the MSW-condition (Figures 4A,B). The first interval corresponded, and likely was due, to the motor behavior the monkeys exhibited only in the MSW-condition. Compared to the SW-condition, the firing rate in the MSW-condition was significantly increased from 500 ms before until 200 ms after bar touch (13% contrast) and significantly decreased from 300 to 1000 ms after the bar touch, both in tone-alone and in noise-tone trials (7 and 8% contrast).

The second interval with significantly different firing rates corresponded to the phasic response to the onset of the first sound in a trial, which was the tone in tone-alone trials and the noise burst in noise-tone trials (10 and 7% contrast). No significant differences were found in the phasic responses to all other sound transients, including the onset of the tone in noise-tone trials. The differences at the first sound were partially resulted from the decreased firing following bar touch in the MSW-condition: Indeed, the relative phasic sound response to the first sound was not significantly different in tone-alone trials.

The third interval with significantly different firing rates overlapped the steady-state part of the first sound when firing rates were significantly lower in the MSW-condition than in the SW-condition (12% contrast in tone-alone trials and 9% contrast in noise-tone trials). Since the steady-state part of the first sound largely overlapped with bar holding, the different firing rates were likely due to bar holding in the MSW-condition (Figure 5).

##### SMW-condition vs. MSMW-condition.

We obtained similar results when we compared the two high-effort conditions SMW and MSMW (Figures 4C,D). Firing rates differed relative to bar touching, bar holding and the steady-state part of the noise burst. There were also no consistent differences in the phasic sound responses between the MSMW- and the SMW-condition. High- and low-effort conditions differed mainly in the 1000 ms interval before initiation of the motor behavior, when baseline firing was significantly increased by 9% from the SMW-condition to the MSMW-condition. The latter difference was likely due to the different motor responses the monkeys made in the two conditions: a bar touch in the SMW-condition and a bar release in the MSMW-condition. In conclusion, except for trivial differences, firing rates did not consistently differ between the self- and externally initiated conditions when conditions with different effort were compared.

#### Comparison of Neuronal Activity Between Conditions With Different Motor Responses

We also performed two comparisons to find neuronal activity that was related to whether or not the monkeys exhibited a motor response to the sounds. We compared two externally-initiated conditions (SMW and SW) and two self-initiated conditions (MSMW and MSW).

##### SW-condition vs. SMW-condition.

The population firing rates of the SW-condition and the SMW-condition differed significantly during baseline (17% contrast; Figures 4E,F). Although differences were also observed a few hundred milliseconds after tone onset, they likely reflected that motor-related firing was only present in the SMW-condition. Aside from these differences, there were no significant firing differences in other time periods, including those with phasic sound responses.

##### MSW-condition vs. MSMW-condition.

Similar to this comparison between a simple and a task condition the comparison between two task conditions revealed no firing differences that were indicative of an interaction between the firing related to the motor and other task components (Figures 4G,H). There were no significant differences in the sound-evoked firing between the MSW-condition and the MSMW-condition. Similarly, baseline firing differed between these conditions even though this was not significant. Although the firing rate was significantly different between the self-initiated conditions during the steady-state part of the tone, this difference could be explained by the firing related to the bar release the monkeys made in the MSMW-condition only. The only significant firing difference between the MSW- and MSMW-condition that could not be explained by superposition of the firings related to the different task components was found relative to the motor behavior by which the monkeys initiated the trial. At this time, the firing rates were higher in the MSMW-condition than in the MSW-condition, both from 500 ms before to 200 ms after bar touch (contrast 10%) and from 300 to 1000 ms after bar touch in tone-alone trials (contrast 10%).

In conclusion, sections Neuronal Activity in the Instrumentally-Trained Monkeys During the Simple Block and Neuronal Activity of the Instrumentally-Trained Monkeys in the Task Block indicated that largest changes in neuronal firing rates occurred when the monkeys received water, either automatically after sound presentation or for correctly handling the touch bar relative to sounds. Changes related to the execution of hand movements were weak or absent.

### Effects of Pairing Sounds With Water in the Passive Monkeys

To find out whether the water-related changes of auditory cortical firing depend on the monkeys’ experience, we performed additional experiments on monkeys with different experience. These passive monkeys were tested with the same three simple conditions as the two instrumentally-trained monkeys but were not trained on the three tasks.

#### Licking Activity Differs Between Simple Conditions

We first established that the two passive monkeys (monkey D and monkey E) discriminated the three simple conditions. Analysis of video recordings from 14 of a total of 18 sessions (9 in monkey D and 5 in monkey E) revealed that the monkeys’ baseline licking activity differed between the three simple conditions (Figures 2E,F). It tended to be lowest in the S-condition, higher in the W-condition and highest in the SW-condition. Although the order of the two latter conditions was opposite to that in the instrumentally-trained monkeys, the conditions did not differ significantly. Similar to the instrumentally-trained monkeys, the licking activity of the passive monkeys varied differently within a trial in the three conditions. While there was no clear variation in the S-condition, variations were present in the W- and SW-condition. The latter conditions differed in the time when the licking activity increased most strongly within a trial, which was later in the W-condition (3560 ms) than in the SW-condition (1820 ms in tone-alone trials and 1740 ms in noise-tone trials). This suggests that also the passive monkeys associated the sounds with water.

#### Neuronal Activity Differs Between the Simple Conditions

To find out how pairing sounds with water altered neuronal firing in the passive monkeys, we analyzed multiunit activity recorded from 75 sites in auditory cortex (37 from monkey D and 38 from monkey E; obtained during 9 sessions in each monkey). BF ranged from 0.14 to 13.36 kHz (median 1.6 kHz) and BW from 1.58 to 7.95 octaves.

Figures 2G,H show the median population firing rates of the passive monkeys for the three simple conditions. Similar to the instrumentally-trained monkeys, pairing sounds with water also resulted in changes of the firing rates in various time intervals of the trials compared to when sounds and water were presented alone (Tables 3, 4).

**TABLE 3:** Median contrasts of the firing rates between experimental conditions of the passive monkeys.

**Component**		**Time interval**	**Start (ms)**	**End (ms)**	**S vs. SW**	**W vs. SW**
B		Baseline	−1700	−700	**57.8**	8.6
S	(T)	Tone – on	0	100	8.0	**95.6**
		Tone – off	0	100	9.6	**39.0**
		Tone – SS	−1900	−700	**44.2**	**19.6**
	(NT)	Noise – on	0	100	15.4	**96.2**
		Noise – off	0	100	**36.1**	**38.6**
		Noise – SS	200	1600	**48.5**	10.8
		Tone – on	0	100	16.5	**93.6**
		Tone – off	0	100	24.1	**46.8**
		Tone – SS	−1900	−700	**42.6**	12.8
W		Water – T	2500	3500	**87.1**	6.0
		Water – NT	2500	3500	**72.1**	8.9

**Results are based on 75 multiunits. Other conventions as in Table 1.**

**TABLE 4:** *P*-values of the comparisons shown in Table 3.

**Component**		**Time interval**	**Start (ms)**	**End (ms)**	**S vs. SW**	**W vs. SW**
B		Baseline	−1700	−700	0.000	0.025
S	(T)	Tone – on	0	100	0.301	0.000
		Tone – off	0	100	0.102	0.000
		Tone – SS	−1900	−700	0.000	0.001
	(NT)	Noise – on	0	100	0.015	0.000
		Noise – off	0	100	0.000	0.000
		Noise – SS	200	1600	0.000	0.032
		Tone – on	0	100	0.112	0.000
		Tone – off	0	100	0.017	0.000
		Tone – SS	−1900	−700	0.000	0.032
W		Water – T	2500	3500	0.000	0.138
		Water – NT	2500	3500	0.000	0.215

Replicating the findings of the instrumentally-trained monkeys, the baseline firing of the passive monkeys increased significantly from the S-condition to the SW-condition (58% contrast). Opposite to the instrumentally-trained monkeys, the baseline firing of the passive monkeys was largest in the SW-condition and not in the W-condition, although none of the comparisons was significant in either group of monkeys. The two groups also differed in the phasic sound responses, which were larger in the SW-condition than in the S-condition in the passive monkeys, even though this was significant only for the weak response to noise burst offset (36% contrast). The reason for the opposite results in the two groups could be that the baseline differed quite strongly between the conditions in the passive monkeys. When we accounted for baseline changes by computing the relative phasic response, we confirmed that these responses were significantly reduced in the SW-condition relative to those in the S-condition in the passive monkeys (contrasts 21–30%). The baseline changes also explain why we found significantly different firing rates during the steady-state part of the tone and the noise burst in the three conditions tested on the passive monkeys. In conclusion, despite some quantitative differences, pairing sound with water had similar effects on the neuronal firing in instrumentally-trained and classically conditioned monkeys.

## Discussion

### Neuronal Firing in Auditory Cortex Related to Individual Components of Auditory Tasks

#### Water Related Firing

The present study confirms and extends the knowledge that neuronal firing in primary auditory cortex can be related to the delivery of water (Brosch et al., 2011b, 2015). In our previous studies, this relationship was obtained by comparing conditions in which monkeys received different amounts of water for correct performance of categorization or audiovisual tasks. In the present study, this relationship was obtained in several other tasks and in even in a condition in which only water was delivered. Moreover, we observed water-related firing in the auditory cortex of monkeys that had undergone classical conditioning of sounds with water. These observations thus indicate that water-related firing can be present in auditory cortex in many behavioral contexts and in subjects with different experiences of how they have learned the relationships between water, sounds and motor behavior. The observation of water-related firing in the W-condition also challenges our earlier observation that auditory cortex neurons do not fire relative to non-auditory components if the components are part of a non-auditory task (Brosch et al., 2005). We note that the current study, together with earlier studies (Brosch et al., 2011b, 2015), increases the number of subjects with water-related firing in auditory cortex from four to eight. A conjunction analysis (Friston et al., 1999) for this number of subjects reveals a quite high probability (0.67) that the individually significant effects are present in the entire population of longtail macaques.

We previously proposed that water-related firing reflected physical properties of the water stimulus, or its reward value (Brosch et al., 2011b). Observations made in the current study, however, favor an alternative interpretation. Because the neuronal firing preceded the monkeys’ licking activity by several hundred milliseconds, we propose that water-related firing is linked to the consumption of water and is triggered by the sight of the water on the spout, by the sounds, or by the execution of the goal-directed hand movement. Our interpretations thus differ in whether water-related firing reflects the (unconditioned) stimulus aspects of the water or the (unconditioned) response aspects of the motor activity by which the monkeys drink water. It is possible that both aspects of water rewards are represented in auditory cortex and that their relative contributions change during a trial even in individual neurons, as observed by us earlier (Brosch et al., 2011b). Future experiments are required to address this issue.

Our study also extends previous accounts that auditory cortex can respond to unconditioned stimuli or can be active during the execution of unconditioned responses not only when aversive stimuli are present but also when appetitive stimuli are present. Letzkus et al. (2011) reported that neuronal firing in auditory cortex changed for several seconds after brief electrical stimulation of the foot. Ide et al. (2013) also showed that electrical foot shocks increased auditory cortical firing rates, the size of which depended on how strongly the foot shock had been associated with a sound.

#### Motor-Related Firing

In the present study we describe that neuronal firing in auditory cortex can be related to two types of motor-activity of subjects. Firstly, there was firing that was related to bar touching. It was characterized by a brief interval with an increased firing rate shortly after bar touch which is followed by a decreased firing rate for at least one second. Secondly, there was firing that was related to the licking activity of the monkeys. These observations extend previous observations of motor-related firing in auditory cortex (Brosch et al., 2005, 2011a, 2015; Yin et al., 2008; Niwa et al., 2012). We note that in addition to these types of motor-activity, which are directed toward obtaining reward, there are also reports that firing in auditory cortex is related to other types of motor activity that was spontaneously expressed by the animals and that obviously was not directed toward reinforcers. These were vocalizations (Eliades and Wang, 2003) and walking on a treadmill (Schneider et al., 2014). It thus appears that auditory cortex can be active during a large variety of audiomotor behaviors in subjects.

### Neuronal Firing in Auditory Cortex When Components of Auditory Tasks Are Combined

#### Baseline Firing

Results of the present study support and extend previous observations that the baseline firing rate in auditory cortex is increased relative to an S-condition when sounds are paired with water or other reinforcing stimuli. This was previously observed for conditions equivalent to those used here, including pairing sound with water (Kitzes et al., 1978) and requiring specific motor behavior to the sounds to receive reinforcement (Scott et al., 2007; Massoudi et al., 2014; Bgur et al., 2018, but see Otazu et al., 2009; Kato et al., 2015). Here, we also showed that relative to the SW-condition, increases in baseline firing rate occurred when a motor response was made to the sound. This suggests that baseline firing rates are increased both when reinforcers are present and when motor responses are executed and that this can occur independently from each other. We observed no consistent decreases of baseline firing when motor behavior preceded and thus triggered sounds relative to conditions in which sounds were externally triggered. The differences in firing rates that were observed shortly before the motor behavior that triggered sounds were likely related to the proper motor behavior and did not reflect the difference between self- and externally generated sounds (Eliades and Wang, 2003; Buran et al., 2014; Carcea et al., 2017).

Our study also indicates that the baseline firing rate in auditory cortex can depend on non-auditory task components, such as in the W-condition in which baseline firing rates were increased relative to the S-condition. The question how the baseline firing rate in the W-condition is related to that in other conditions was not resolved by the present study and thus requires further experiments. These may also clarify why we obtained different results in the instrumentally-trained and the passive monkeys and why other studies found effects of self-initiation in conditions without reinforcement (Müller-Preuss and Ploog, 1981; Eliades and Wang, 2003; Schneider et al., 2014).

#### Sound-Evoked Firing

In the present study, combining sounds with water or requiring motor behavior relative to sounds resulted in changes of both the phasic and the tonic sound responses in auditory cortex. This is compatible with earlier studies which also analyzed equivalent conditions; an S-condition with either an SW-condition (Kitzes et al., 1978; Letzkus et al., 2011), an MS-condition (Eliades and Wang, 2003), or conditions composed of S-, M, and W-components (Beaton and Miller, 1975; Benson and Hienz, 1978; Kitzes et al., 1978; Scott et al., 2007; Otazu et al., 2009; Letzkus et al., 2011; Massoudi et al., 2014; Schneider et al., 2014; Kato et al., 2015; Carcea et al., 2017). Our finding that the changes in phasic and tonic sound responses were largely independent of each other may be of particular importance because this may reconcile conflicting results of the direction of the task effects in the earlier studies. Sorting these studies according to response time reveals that phasic responses were decreased (Beaton and Miller, 1975; Benson and Hienz, 1978; Otazu et al., 2009; Schneider et al., 2014; Carcea et al., 2017) whereas tonic responses were increased or not changed (Kitzes et al., 1978; Scott et al., 2007; Letzkus et al., 2011; Massoudi et al., 2014; Kato et al., 2015).

In the instrumentally-trained monkeys, the phasic sound responses were clearly different when we compared the S-condition to the SW-condition (up to 25%). By contrast, the phasic sound responses did not change, or changed very little and then changed inconsistently when we compared the SW-condition to the task conditions: If present, changes were limited to the first sound transient in a trial and disappeared if baseline changes were taken into account (e.g., for the comparison between the SW-condition and MSW-condition). These observations, thus, suggest that if an S-condition is compared to a task condition, the phasic sound responses are most strongly affected by the presence of water or some other reinforcing stimulus in the task conditions rather than by the subjects’ execution of goal directed movements relative to sounds.

In the present study, tonic sound responses were, at most, slightly changed across conditions. One reason for this could be that already during the S-condition there was only weak evidence for tonic sound responses. Hence, it is possible that the changes in the steady-state firing rates simply reflect differences in baseline firing which continued throughout the presentation of the noise burst, or reflect that the firing rate was decreased after bar touch and increased after bar release.

It may be counterintuitive that phasic responses to sounds are suppressed when sounds are paired with water or when motor responses have to be executed to the sounds because the sounds are behaviorally more relevant in the task conditions and the SW-condition than in the S-condition. Indeed, responses to sounds with a positive value were found to be enhanced (David et al., 2012). However, such a view does not take into account that during task performance, auditory cortex neurons are not solely activated by sounds but are also activate relative to non-auditory task components (such as the water). Thus, selective suppression of sound-evoked firing may be considered as an efficient mechanism of gain control and metabolic energy saving that keeps the total numbers of spikes fired by neurons constant relative to different task components.

### Which Mental Processes Affect Neuronal Firing in Auditory Cortex?

Our approach of comparing conditions with different number of task components allows us to conjecture which mental processes are reflected in auditory cortex. We also note that such discussion depends on the concepts the researchers have of a given mental process and on the cognitive strategies utilized by individual animals during task performance.

The present study found that the sound-evoked responses changed from the S-condition to the SW-condition and the other task conditions. Because this was found between the S-condition and the SW-condition our study supports the view that auditory cortex is involved in the formation of associations between sounds and water (see also Brosch et al., 2011a). By contrast, there were not clear and consistent changes of sound-evoked responses when the SW-condition was compared to conditions in which the monkeys had to respond to sound or triggered sound by some motor behavior. This suggests that our findings do not support the view that auditory cortex is involved in the formation of associations between sounds and motor actions, which is at variance with other findings from other studies (Vaadia et al., 1982; David et al., 2012; Jaramillo et al., 2014; Huang et al., 2019) and which merits further studies.

Our finding that the sound-evoked responses changed from the S-condition to the SW-condition and the task conditions is also compatible with the proposal that activity in auditory cortex reflects the value of sounds (Kitzes et al., 1978; Rutkowski and Weinberger, 2005; Letzkus et al., 2011; David et al., 2012). Our finding that no further changes in phasic sound responses occurred when the SW-condition was compared to the task conditions suggests that the representation of the value of sound in auditory cortex only requires the presence of a reinforcer but is independent of the task requirement.

We consider that our observations support the view that auditory cortical activity is related to the general activity level, or arousal, of animals. In our study the level of arousal was estimated from the monkeys’ motor activity. It likely changed from the S-condition, where the monkeys most of the time sat quietly but awake in the primate chair, to the SW-condition, where the monkeys had increased licking activity because of the water, to the three task conditions, where the monkeys also had to touch and release a bar at time points determined by the experimenters. The changes of arousal between these conditions were paralleled by changes in the baseline firing in auditory cortex. A correspondence between auditory cortical activity and arousal has been proposed in earlier studies (Schneider et al., 2014; Zhou et al., 2014; Williamson et al., 2015) and, e.g., was reflected in finding that the spontaneous firing in mouse auditory cortex is correlated with the animals’ pupil diameter (McGinley et al., 2015). The pupil diameter is a physiological parameter that is generally considered to reflect activity of neuromodulatory system and the arousal level of animals (Bradley et al., 2008). We note that the difference in licking activity between the S-condition and the SW-condition were also paralleled by a change in the sound-evoked responses. Thus, the difference in sound-evoked responses and baseline firing indicates that auditory cortical activity is also shaped by the level of arousal.

By contrast, our findings are not consistent with the view that activity in auditory cortex is related to the efforts subjects deploy on sound processing (Fritz et al., 2003; Atiani et al., 2009). This is inferred from our comparisons between conditions in which subjects received water either automatically after the presentation of sound or had to make a motor response to sound, and this was found both when a simple condition was compared to a task condition and when two task conditions were compared. Likewise, our findings do not support the view that activity in auditory cortex is related to sensing agency, i.e., to the ability to distinguish sounds that are produced by own bodily movements from sounds that are caused by a change in the environment (Eliades and Wang, 2003; Otazu et al., 2009; Carcea et al., 2017). This issue was addressed by comparing trials that were either initiated by a computer or by the subjects themselves, and both when the effort was low (SW vs. MSW) or high (SMW vs. MSMW). Further experiments are required to address the question whether the opposite findings are due to species differences or to methodological differences.

## Data Availability Statement

The datasets generated for this study are available on request to the corresponding author.

## Ethics Statement

The animal study was reviewed and approved by the Animal Care and Ethics Authority of the State of Saxony-Anhalt [Referat Verbraucherschutz, Veterinärangelegenheiten Landesverwaltungsamt Sachsen-Anhalt Dessauer Straße 70 06118 Halle (Saale)].

## Author Contributions

SK designed the study, acquired, analyzed, and interpreted the data, and wrote the manuscript. MB designed the study, interpreted the data, and wrote the manuscript. ES and AG acquired and interpreted the data, and wrote the manuscript. FO interpreted the data and wrote the manuscript.

## Conflict of Interest

The authors declare that the research was conducted in the absence of any commercial or financial relationships that could be construed as a potential conflict of interest.
